# Enzymatic Synthesis of a Novel α‐Linolenic Acid‐Enriched Phytoceramide From Perilla Seed Oil and Its Multifunctional Bioactivity in Skin Barrier Repair and Anti‐Aging

**DOI:** 10.1002/cbdv.71808

**Published:** 2026-10-11

**Authors:** Jumao Yuan, Ning Hao, Borui Shi, Zhenzhen Wang, Jie Yu, Nuo Shi, Lin Lin

**Affiliations:** ^1^ Peterson's Lab Shanghai P.R. China; ^2^ College of Biotechnology and Pharmaceutical Engineering State Key Laboratory of Materials‐Oriented Chemical Engineering Jiangsu National Synergetic Innovation Center for Advanced Materials (SICAM) Nanjing Tech University Nanjing P.R. China; ^3^ Baudry Biotech. Co., Ltd Nanjing Jiangsu P.R. China; ^4^ Institute of Symbolcell Biotechnology Nanjing Jiangsu P.R. China; ^5^ Department of Emergency Ruijin Hospital, Shanghai Jiaotong University School of Medicine Shanghai P.R. China

**Keywords:** anti‐aging, enzymatic synthesis, perilla seed oil, phytoceramide, skin barrier, transcriptomics

## Abstract

Ceramides are essential lipid components of the skin barrier, accounting for approximately 50% of intercellular lipids in the stratum corneum. Their deficiency is associated with various skin disorders, including atopic dermatitis and xerosis. This study aimed to enzymatically synthesize a novel phytoceramide from perilla seed oil (PSO‐Cer) and evaluate its multifaceted bioactivity, including skin barrier enhancement and anti‐aging effects, in comparison with oleic acid‐derived Ceramide IIIB. PSO‐Cer was synthesized enzymatically and characterized by HPLC‐CAD and LC‐QTOF. Its biological effects were assessed in human keratinocytes (HaCaT) and dermal fibroblasts‐adult (HDF‐a) through cytotoxicity assays, qPCR of genes related to barrier function and extracellular matrix (ECM) synthesis, western blot, and transcriptome sequencing. HPLC‐CAD confirmed PSO‐Cer was rich in polyunsaturated fatty acid chains, notably α‐linolenic acid (34.49%). Both PSO‐Cer and Ceramide IIIB were non‐cytotoxic up to 25 µg/mL and significantly upregulated genes critical for epidermal barrier function (FLG, IVL, TGM1, CERS3, and ELOVL4) and ECM components (COL1A1, COL3A1, and ELN) in UVB‐irradiated fibroblasts. Transcriptome analysis revealed that while both ceramides shared common mechanisms, namely activation of mTOR signaling and sphingolipid metabolism, PSO‐Cer exhibited distinct properties, positioning it as a promising multifunctional active for advanced skincare formulations aimed at comprehensive skin health.

## Graphical abstract

An enzymatically synthesized α‐linolenic acid‐enriched phytoceramide from perilla seed oil (PSO) is presented. This novel ceramide demonstrates potent activation of skin barrier genes in keratinocytes and protection against UVB‐induced extracellular matrix (ECM) degradation in fibroblasts. Transcriptomic analysis reveals concurrent upregulation of antioxidant pathways and suppression of cellular senescence, distinguishing it from conventional ceramides. This multifunctional ceramide represents a sustainable, plant‐derived active for comprehensive skin health.

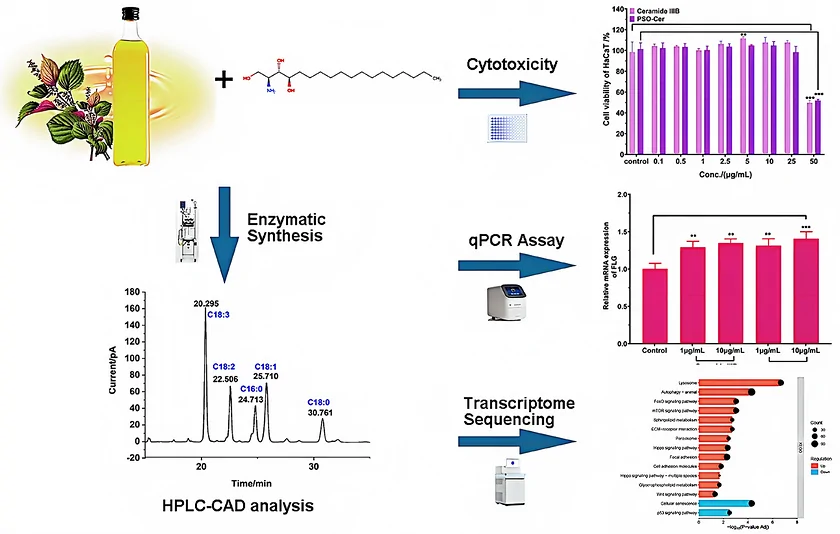

## 1 Introduction

As the largest organ of the human body, with its outermost layer, the stratum corneum, the skin forms a physical barrier that defends against external environmental stimuli and prevents the loss of internal moisture [1]. In this “brick‐and‐mortar” structure, the orderly arrangement of keratinocytes (“bricks”) and intercellular lipids (“mortar”) is crucial for maintaining barrier function [2]. Among these intercellular lipids, ceramides, as the most abundant and functionally vital components, account for approximately 50% of the total intercellular lipids, not only providing structural support but also playing a central role in regulating skin hydration, anti‐inflammation, antioxidation, and signal transduction [3, 4, 5].

The synthesis of ceramides is a complex biological process involving a series of synthases, such as serine palmitoyltransferase (SPT), ceramide synthases (CERS), and fatty acid elongases (ELOVL) [6]. Among these, CERS3 and ELOVL4 are crucial for the production of ultra‐long‐chain ceramides, which are vital for the skin barrier [7, 8]. Simultaneously, the normal expression of terminal differentiation markers in keratinocytes, such as filaggrin (FLG), involucrin (IVL), and transglutaminase (TGM), is a prerequisite for forming the complete cornified envelope and cross‐linking ceramide precursors [9]. Any factors that lead to aberrant expression of these key proteins or enzymes, such as UV radiation, oxidative stress, aging, or genetic defects, probably disrupt ceramide homeostasis, trigger skin barrier dysfunction, and are closely associated with a range of dermatological conditions including atopic dermatitis, psoriasis, dry skin, and photoaging [1, 10]. PSO‐Cer is structurally distinct from previously reported plant‐derived ceramides. Typical plant‐derived ceramides—such as those from rice, wheat, *Camellia japonica*, *Prunus mume*, and *Sesamum indicum*—contain predominantly saturated or mono‐unsaturated fatty acyl chains (primarily C16:0 and C18:1) [11, 12]. In contrast, PSO‐Cer is notably enriched in polyunsaturated C18:3 (α‐linolenic acid, 34.49%), a characteristic inherited directly from its perilla seed oil (PSO) precursor. This high degree of unsaturation is hypothesized to confer additional antioxidant and anti‐inflammatory benefits beyond basic barrier repair. Furthermore, PSO represents a renewable, non‐animal source with established agricultural production, offering sustainability advantages over animal‐derived or chemically synthesized ceramides. The structural distinctiveness of PSO‐Cer, combined with the sustainability of its source, formed the basis for investigating its differentiated biological activity.

However, ceramides used in traditional skincare products are mostly derived from animal extraction or chemical synthesis, which pose issues such as limited sources, potential sensitization, and structural mismatch with human native ceramides [13]. Consequently, developing a new source of ceramides that is safe, efficient, and highly compatible with human skin has become a research focus. Plant‐derived ceramide precursors, such as PSO, are natural plant oils rich in α‐linolenic acid and various other unsaturated fatty acids. The potential applications of phyto‐derived ceramides in improving epidermal barrier function have attracted increasing attention [14]. Recent studies have verified their prominent anti‐inflammatory and antioxidant properties [15, 16]. More importantly, it may serve as a high‐quality plant‐based raw material for synthesizing ceramides. So far, there is a lack of systematic research on the specific synthesis pathways of ceramides derived from PSO, their regulatory effects on skin barrier‐related genes, and a comparison of their efficacy as well as molecular mechanisms with those of commonly marketed ceramides, such as oleic acid‐derived ceramide. Ceramide IIIB (N‐oleoyl‐phytosphingosine) was selected as the comparator because it is one of the most widely used ceramide actives in commercial cosmetic formulations, particularly for sensitive and dry skin applications. While not naturally found in human skin, Ceramide IIIB has demonstrated beneficial effects in barrier repair and moisturization in multiple clinical and in vitro studies. Its established efficacy and market prevalence make it an appropriate benchmark for evaluating the performance of the novel PSO‐Cer. To date, no systematic study has investigated the specific synthesis pathways of ceramides derived from PSO, their regulatory effects on skin barrier‐related genes, or their comparative efficacy and molecular mechanisms against commonly marketed ceramides. Therefore, the present study aims to investigate the synthesis and biological efficacy of PSO‐derived phytoceramide (PSO‐Cer). Through characterizing its composition using high‐performance liquid chromatography with charged aerosol detection (HPLC‐CAD) and systematically evaluating the efficacies from the aspects of antioxidant, anti‐cellular senescence, and barrier repair, two models of immortalized human keratinocytes (HaCaTs) and human dermal fibroblasts (HDFs) were utilized in in vitro experiments. Meanwhile, in order to delve into the mechanisms, quantitative PCR (qPCR) was conducted to assess its regulatory effects on key gene expression in comparison with common oleic acid‐derived ceramide (Ceramide IIIB). Especially, this approach highlighted their distinct properties in modulating critical pathways such as the peroxisome pathway, FoxO signaling pathway, p53 signaling pathway, cellular senescence pathway, focal adhesion, and glycerophospholipid metabolism [17, 18]. As a result, these findings provided a solid theoretical foundation and experimental data support for developing highly effective PSO‐Cer‐based products in skin care.

## 2 Methods

### 2.1 Synthesis of PSO‐Cer

Enzymatic ceramide synthesis represents an environmentally friendly and efficient alternative to conventional chemical methods. Different from traditional routes that depended on energy‐intensive acid chlorides and generated salt waste, this approach employed natural oils as acyl donors and operated under mild conditions [19]. The key point was the high selectivity of lipases such as Novozym 435, which catalyzed amide formation with precision, minimizing byproducts, and simplifying purification [20, 21]. The use of solvents and reusable immobilized enzymes further enhanced sustainability while reducing costs [22]. By enabling direct utilization of natural oils, the enzymatic method provided an eco‐friendly and sustainable route to bio‐derived ceramides, fully aligning with green chemistry principles.

The enzymatic synthesis followed the optimized protocol established by Zhang et al. [23], who demonstrated that Novozym 435 in cyclopentyl methyl ether (CPME) at 65°C with a substrate molar ratio of 1:1 achieves 94% yield for ceramide IIIB. The reaction proceeds via a two‐step mechanism: (1) lipase‐catalyzed hydrolysis of triacylglycerols in PSO to release free fatty acids, and (2) lipase‐catalyzed amidation between phytosphingosine and the released fatty acids. This two‐step pathway is well‐documented for lipases like Novozym 435 in the presence of amino alcohols and water [19, 20, 21]. Phytosphingosine (>90% Purity, Big Bang Hunan Biological Pharmaceutical Co., Ltd., China) and PSO (>50% α‐Linolenic Acid, Baolong Zhongcheng Shanghai Biotechnology Co., Ltd., China) were charged into the reactor (Jacketed Glass Reactor, Shanghai Yuanheng Instrument Co., Ltd., China) at a molar ratio of 1:0.33∼0.4 (phytosphingosine to oil). The mixture was pre‐incubated at 65°C for 1 h under stirring to ensure complete dissolution. Novozym 435 (from Candida antarctica, Novozymes) was then added at a loading of 30 wt% relative to phytosphingosine mass. The reaction proceeded at 65°C for 20–24 h with continuous agitation. The process was monitored by thin‐layer chromatography (TLC) (TLC Silica gel 60 F_254_, Merck KGaA) at 2‐h intervals using chloroform:methanol = 10:1 as the mobile phase (phytosphingosine Rf = 0.15; ceramide product Rf = 0.65); the reaction was allowed to proceed until the phytosphingosine spot had completely disappeared, as previously described by Zhang et al. [23]. After completion, the immobilized enzyme was recovered by filtration for reuse, and the CPME solvent was evaporated under reduced pressure (Rotavapor R‐300, BUCHI Labortechnik AG, Switzerland). The crude product was purified by silica gel column chromatography (Isolera Prime, Biotage) using a gradient of chloroform:methanol (from 50:1 to 10:1). The isolated yield was >90%, consistent with the 94% yield reported by Zhang et al. [23], and the purified product showed >95% purity by HPLC‐CAD area normalization. The overall synthesis workflow is illustrated in Figure 1.

**FIGURE 1:**
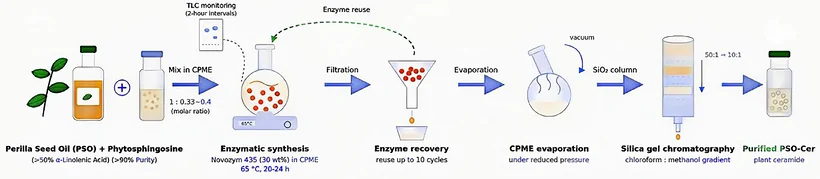
Synthesis and characterization workflow of PSO‐Cer.

### 2.2 Quantitative Characterization

The chromatographic analysis was performed using a Capcell Pak C18 MGII column (250 × 4.6 mm, 3 µm) maintained at 30°C in HPLC (UltiMate 3000, Thermo Fisher Scientific (China) Co., Ltd.). The mobile phase consisted of 0.05% trifluoroacetic acid (Analytical Grade, Shanghai Macklin Biochemical Technology Co., Ltd., China) in water (Phase A, Ultrapure Water, self‐prepared) and methanol (Phase B, HPLC Grade, Tianjin Concord Technology Co., Ltd., China) with a gradient elution program as follows: initial 15% A/85% B held for 5 min, linearly changed to 2% A/98% B at 10 min, maintained until 50 min, then returned to initial conditions at 50.1 min and re‐equilibrated until 60 min. The flow rate was constant at 1.0 mL/min, the injection volume was 10 µL, and the nebulizer temperature of the CAD (Corona Veo, Thermo Fisher Scientific (China) Co., Ltd.) was set at 50°C. For sample preparation, exactly 0.1 g of the sample PSO‐Cer was dissolved in methanol via sonication (KQ‐500DE, Kunshan Ultrasonic Instruments Co., Ltd., China) and diluted to 100 mL to obtain a 1.0 g/L test solution. Analysis was conducted by injecting both blank and sample solutions, with chromatographic data processed using the area normalization method while excluding solvent peaks and minor peaks contributing less than 0.2% of total area. The representative HPLC‐CAD chromatogram is shown in Figure 2, and the quantitative compositional data are presented in Table S2. For structural confirmation, the purified PSO‐Cer was subjected to liquid chromatography‐quadrupole time‐of‐flight mass spectrometry (LC‐QTOF) analysis in positive ion mode. The mobile phase consisted of 0.1% formic acid in water (A) and acetonitrile (B) with a gradient elution. High‐resolution mass spectra were acquired on a SCIEX X500R QTOF system. Collision‐induced dissociation (CID) fragmentation was performed to confirm the ceramide structures. The identified species are summarized in Table 1, with representative fragmentation spectra shown in Figure S1.

**FIGURE 2:**
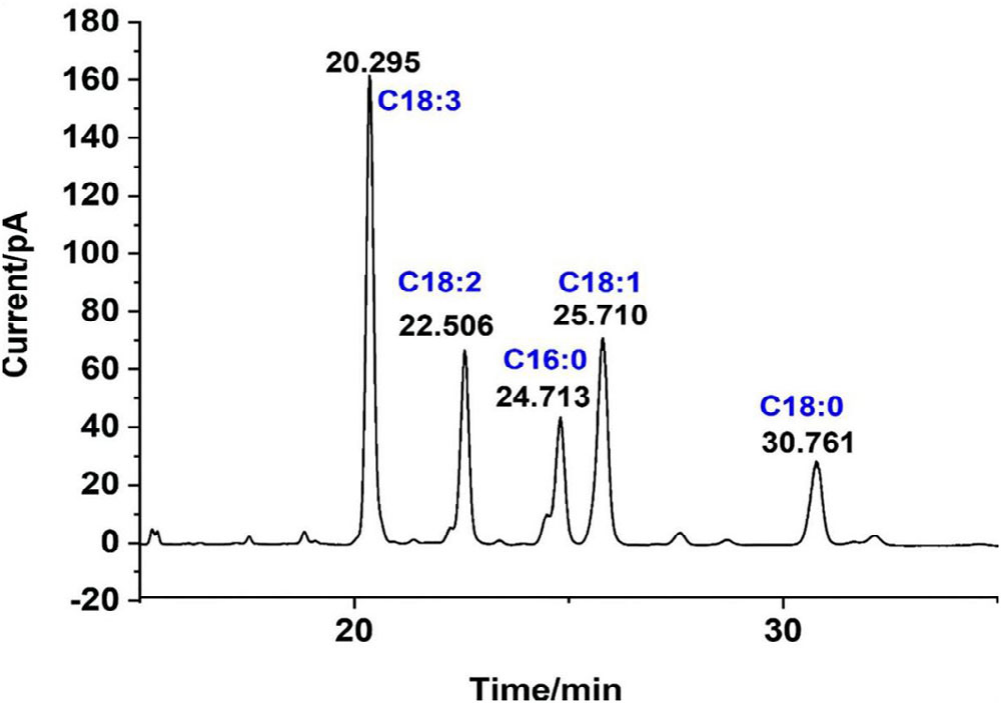
HPLC‐CAD chromatogram of enzymatically synthesized PSO‐Cer.

**TABLE 1:** Major ceramide species identified by LC‐QTOF analysis with characteristic fragment ions.

Ceramide species	Observed *m/z*	RT (min)	Adduct	Mass error (ppm)	Characteristic fragment ions (*m/z*)	HPLC‐CAD (%)
C18:3 (α‐linolenic) ceramide	578.5119	30.96	[M+H]^+^	−5.0	560.50 [M+H−H_2_O]^+^, 282.28 [t18:0−2H_2_O+H]^+^, 261.22 [C_17_H_29_O]^+^	34.49
C18:2 (linoleic) ceramide	580.5290	41.57	[M+H]^+^	−2.4	562.52 [M+H−H_2_O]^+^, 282.28 [t18:0−2H_2_O+H]^+^	17.25
C18:1 (oleic) ceramide	582.5438	41.76	[M+H]^+^	−3.9	564.53 [M+H−H_2_O]^+^, 282.28 [t18:0−2H_2_O+H]^+^	21.89
C16:0 (palmitic) ceramide	556.5270	36.55	[M+H]^+^	−6.1	538.52 [M+H−H_2_O]^+^, 282.28 [t18:0−2H_2_O+H]^+^	12.48
C18:0 (stearic) ceramide	—	—	—	—	Not detected	9.46

### 2.3 Cell Viability

The cytotoxicity of PSO‐Cer and Ceramide IIIB was evaluated on human immortalized keratinocyte cells (HaCaT, Keygen Biotech, Nanjing, China, RRID: CVCL_0038) and human dermal fibroblast cells (HDF‐a, Jennio Biotech, Guangzhou, China, Catalog No. JNO‐H0613; no RRID available in Cellosaurus database) using the Cell Counting Kit‐8 (CCK‐8, Beyotime Biotech, China) assay. Briefly, cells were seeded in 96‐well plates (Corning, USA) at a density of 8000 cells per well and cultured overnight in high‐glucose Dulbecco's Modified Eagle Medium (DMEM) medium (Gibco, USA) supplemented with 10% heat‐inactivated fetal bovine serum (FBS) (Hyclone, New Zealand) at 37°C with 5% CO_2_. After adherence, the cells were treated with either PSO‐Cer or Ceramide IIIB (≥ 98% Purity, Big Bang Hunan Biological Pharmaceutical Co., Ltd., China) at concentrations of 0.1, 0.5, 1, 2.5, 5, 10, 25, and 50 µg/mL in dimethyl sulfoxide (DMSO), and a blank control (culture medium only). Following 24 h of incubation, 10 µL of CCK‐8 solution was added to each well, and the plates were further incubated for 1 h. Optical density was measured at 450 nm using a multifunctional microplate reader (Molecular Devices, USA). Cell viability was expressed as a percentage relative to the blank control group. The cytotoxicity results are presented in Figure 3.

**FIGURE 3:**
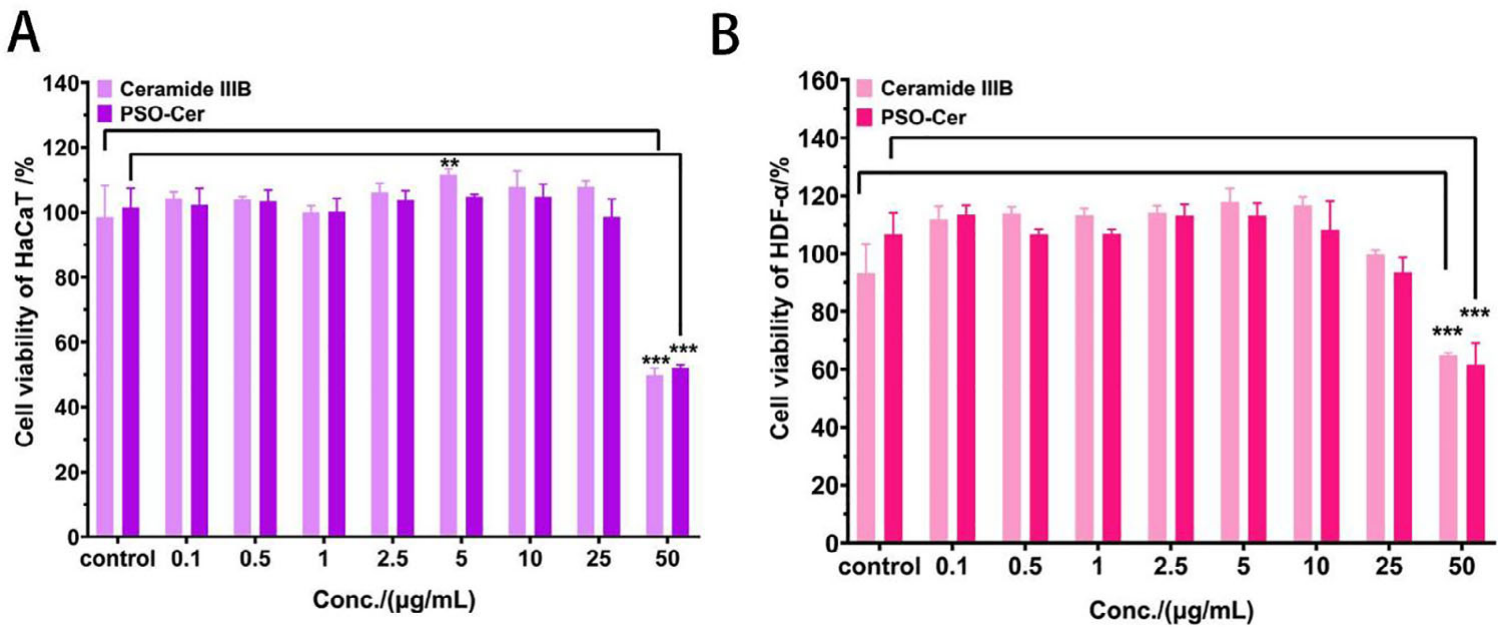
Cytotoxicity assessment of PSO‐Cer and Ceramide IIIB in HaCaT (A) and HDF‐a (B) cells. Cell viability was determined by CCK‐8 assay after 24 h treatment. Data are presented as mean ± SD (*n* ≥ 3). ^***^ *p* < 0.001 versus control.

### 2.4 qPCR Assay

Total RNA was extracted from HaCaT and HDF‐a using TRIzol reagent (Cat. No. 15596026, Thermo Fisher Scientific, USA). RNA integrity was verified using an Agilent 2100 Bioanalyzer (Agilent Technologies, USA), and only samples with an RNA Integrity Number (RIN) > 7.0 were processed. cDNA was synthesized from 1 µg of total RNA using the ReverTra Ace qPCR RT Master Mix with gDNA Remover kit (Code No. FSQ‐301, Toyobo, Japan) according to the manufacturer's instructions. Quantitative real‐time PCR (qPCR) was performed using the SYBR Green Real‐time PCR Master Mix kit (Code No. QPK‐201, Toyobo, Japan) on a QuantStudio 5 Real‐Time PCR System (Thermo Fisher Scientific, USA). The 20 µL reaction mixture contained 10 µL of SYBR Green Master Mix, 2 µL of cDNA template, as well as 0.4 µM of each specific forward and reverse primer. The thermal cycling conditions were as follows: initial denaturation at 95°C for 3 min, followed by 40 cycles of 95°C for 15 s and 60°C for 30 s. Gene expression levels were normalized to the housekeeping gene GAPDH and calculated using the 2–ΔΔCt method [9, 24]. All primer sequences used in the research are listed in Table S4.

The mRNA levels of crucial genes involved in skin barrier and extracellular matrix (ECM) functions were assessed in HaCaT cells and HDF‐a cells. Briefly, cells were irradiated with UVB light at a wavelength of 311 nm and a dose of 300 mJ/cm^2^ using a Philips TL20W/12 RS UVB lamp. This dose is widely used in photoaging research to induce sublethal cellular stress and suppress ECM gene expression in fibroblasts while maintaining cell viability above 80%, modeling chronic UVB exposure relevant to cumulative sun damage [15, 16]. Following irradiation, the cells were treated with either the test compounds or phosphate‐buffered saline (PBS) as a vehicle control and co‐incubated for 24 h. Total RNA was then extracted, and the mRNA expression levels were quantitatively analyzed by RT‐qPCR. The qPCR results for barrier‐related genes in HaCaT cells and ECM genes in UVB‐irradiated HDF‐a cells are presented in Figure 4A–J.

**FIGURE 4:**
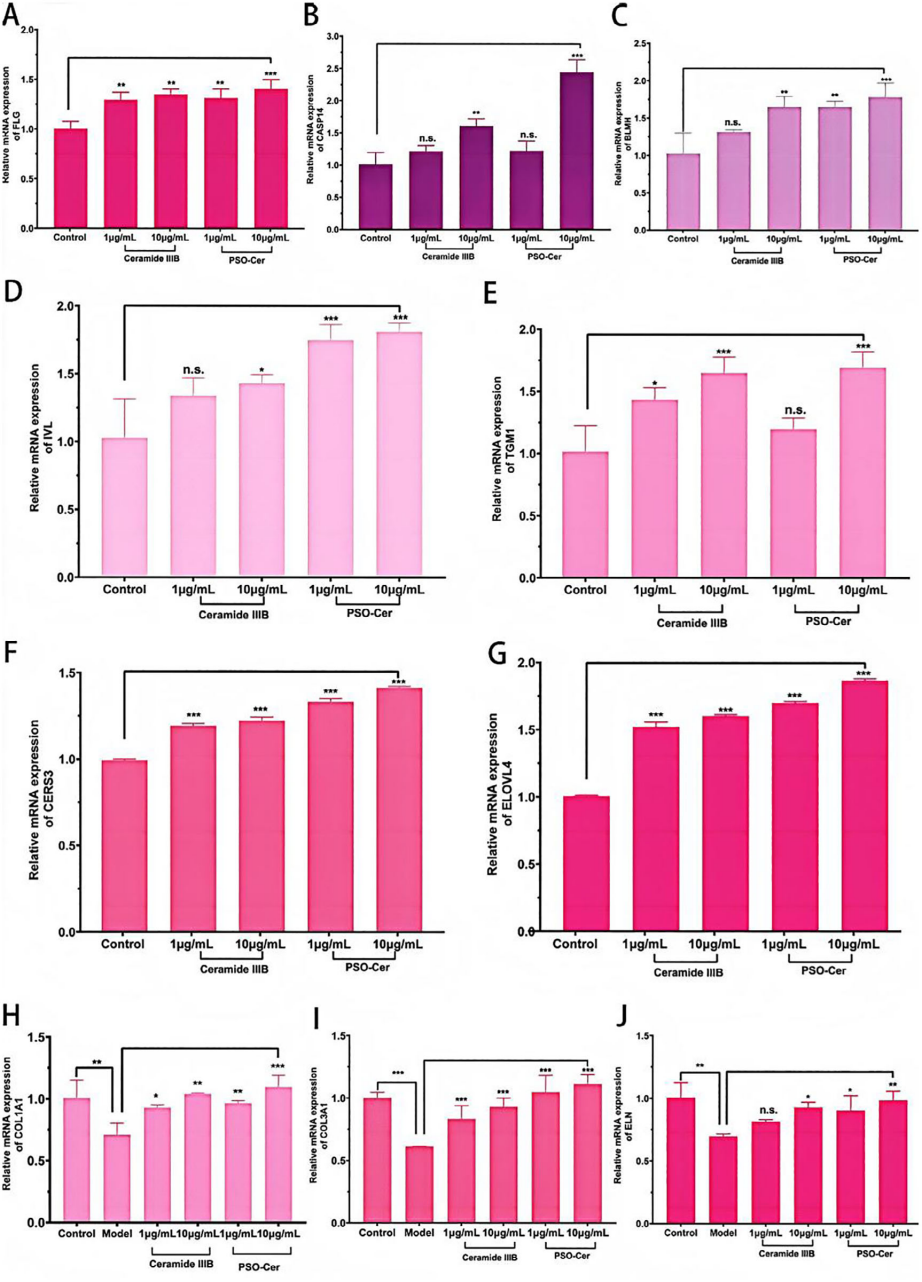
Effects of PSO‐Cer and Ceramide IIIB on mRNA expression of genes related to NMF (A–C), cornified envelope (D,E), and ceramide synthesis (F,G) in HaCaT cells, as well as on ECM components (H–J) in UVB‐irradiated HDF‐a cells. Gene expression was analyzed by qPCR after 24 h of treatment. For HaCaT cells, expression levels of FLG, CASP14, and BLMH (A–C), IVL and TGM1 (D,E), and CERS3 and ELOVL4 (F,G) were assessed. For HDF‐a cells, a cellular senescence model was established by UVB irradiation (300 mJ/cm^2^), after which cells were treated with ceramides for 24 h, and the expression of COL1A1, COL3A1, and ELN (H–J) was evaluated. Data are presented as mean ± SD (*n* = 3). ^*^ *p* < 0.05, ^**^ *p* 0.01, ^***^ *p* 0.001 versus control group (HaCaT) or non‐irradiated control group (HDF‐a); ^*^ *p* 0.05, ^**^ *p* < 0.01, ^***^ *p* < 0.001 versus UVB‐irradiated model group.

### 2.5 Western Blot Analysis

Total protein was extracted from HaCaT and HDF‐a cells using radioimmunoprecipitation assay (RIPA) lysis buffer (Servicebio, China) containing protease inhibitors. Protein concentration was determined using a bicinchoninic acid (BCA) protein assay kit (Servicebio, China). Equal amounts of protein (20 µg) were separated by sodium dodecyl sulfate–polyacrylamide gel electrophoresis (SDS–PAGE) and transferred onto polyvinylidene fluoride (PVDF) membranes (0.45 µm, Servicebio, China). Membranes were blocked with 5% non‐fat milk for 30 min at room temperature and incubated with primary antibodies overnight at 4°C. The primary antibodies used were: anti‐FLG (DF13653, 1:1000, Affinity), anti‐CASP14 (28136‐1‐AP, 1:1000, Proteintech), anti‐IVL (28462‐1‐AP, 1:1000, Proteintech), anti‐TGM1 (12912‐3‐AP, 1:1000, Proteintech), anti‐ELOVL4 (55023‐1‐AP, 1:500, Proteintech), anti‐CERS3 (ab169259, 1:1000, Abcam), anti‐COL1A1 (GB11022, 1:1000, Servicebio), anti‐ELN (15257‐1‐AP, 1:1000, Proteintech), and anti‐ACTIN (GB15003, 1:3000, Servicebio). After washing with Tris‐buffered saline with Tween 20 (TBST), membranes were incubated with horseradish peroxidase (HRP)‐conjugated secondary antibodies (Servicebio, China) for 30 min at room temperature. Protein bands were visualized using enhanced chemiluminescence (ECL) substrate (Servicebio, China) and imaged with a chemiluminescence imaging system (SCG‐W3000, Servicebio, China). Densitometric analysis was performed using AIWBwell software (Servicebio, China), and target protein expression was normalized to ACTIN. The representative western blot bands and densitometric quantification are shown in Figure 5. Raw densitometric data are provided in Table S3A,B. A summary of the consistency between qPCR and Western blot results is presented in Table 3.

**FIGURE 5:**
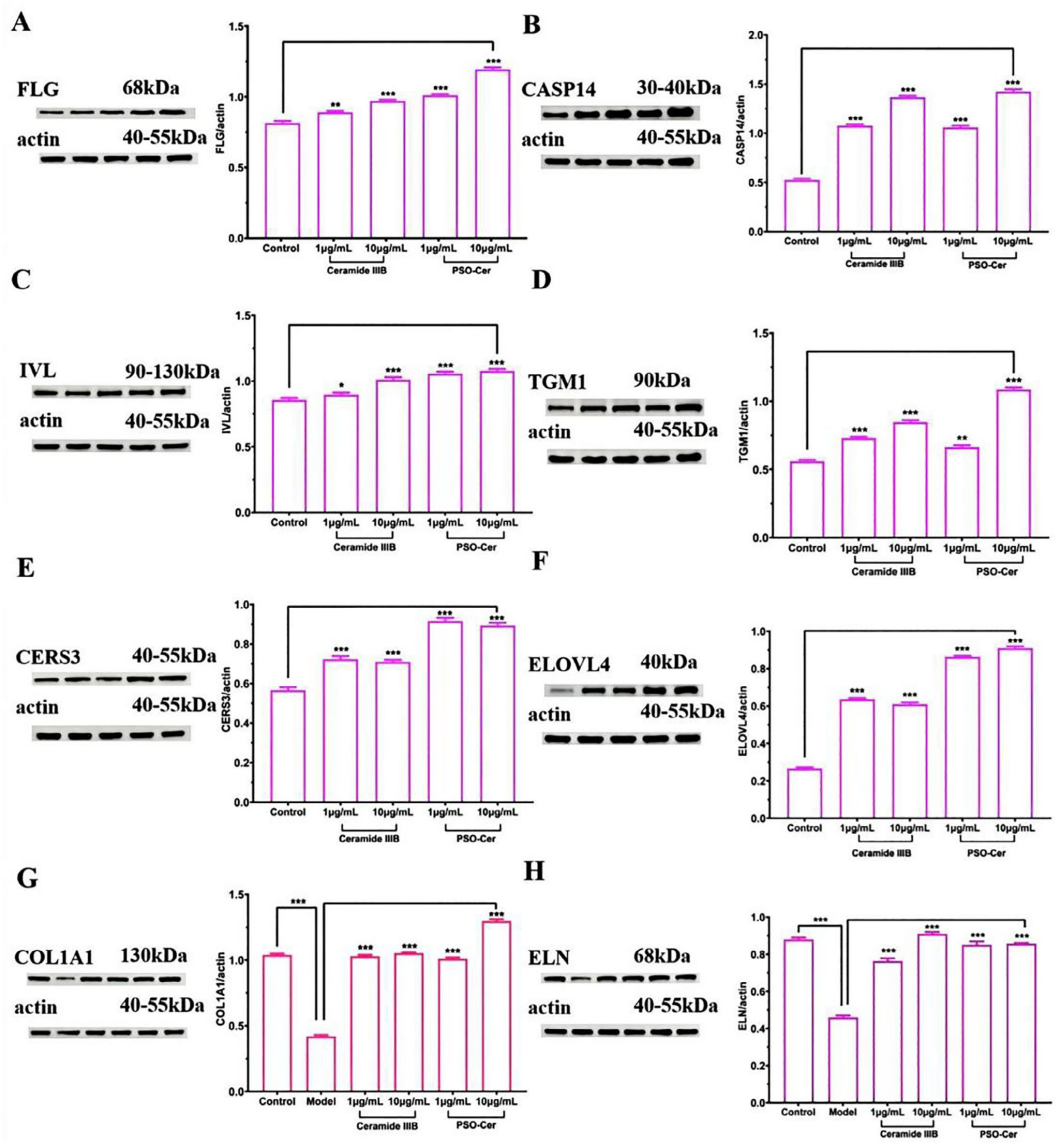
Western blot analysis of protein expression in HaCaT keratinocytes and HDF‐a fibroblasts. (A–F) Representative Western blot bands and densitometric quantification of barrier‐related proteins in HaCaT cells treated with PSO‐Cer and Ceramide IIIB at 1 and 10 µg/mL for 24 h: (A) FLG, (B) CASP14, (C) IVL, (D) TGM1, (E) ELOVL4, and (F) CERS3. (G,H) Representative Western blot bands and densitometric quantification of ECM proteins in HDF‐a fibroblasts subjected to UVB irradiation (311 nm, 300 mJ/cm^2^) followed by 24 h treatment with PSO‐Cer and Ceramide IIIB at 1 and 10 µg/mL: (G) COL1A1 and (H) ELN. Protein expression levels were normalized to ACTIN. Data are presented as mean ± SD (*n* = 3). ^*^ *p* < 0.05, ^**^ *p* < 0.01, ^***^ *p* < 0.001 versus Control (HaCaT) or versus Model (HDF‐a). Full densitometric data are provided in Table S3A,B.

### 2.6 Transcriptome Sequencing

Total RNA was isolated from HaCaT and HDF‐a cells treated with PSO‐Cer and Ceramide IIIB (>95%, Big Bang (Hunan) Biological Pharmaceutical Co., Ltd., China) using TRIzol reagent (Cat. No. 15596026, Thermo Fisher Scientific, USA). RNA integrity and concentration were assessed with an Agilent 2100 Bioanalyzer (Agilent Technologies, USA) and a NanoDrop One/OneC Microvolume UV–vis Spectrophotometer (Thermo Fisher Scientific, USA), respectively. Sequencing libraries were constructed following the standard protocol of the Illumina TruSeq Stranded mRNA LT Sample Preparation Kit (Illumina, USA). The prepared libraries were quantified using a Qubit 2.0 Fluorometer (Thermo Fisher Scientific, USA) and qualified using an Agilent 4200 Tapestation (Agilent Technologies, USA), then subjected to paired‐end sequencing (PE150) on an Illumina NovaSeq 6000 platform (Illumina, USA).

Raw sequencing reads were processed to obtain clean data by removing adapters and low‐quality sequences using fastp software (v0.23.2). The clean reads were then aligned to the human reference genome (GRCh38.p13) using the STAR aligner (v2.7.10a) [25]. Gene expression levels were quantified as FPKM (Fragments Per Kilobase of transcript per Million mapped reads) using featureCounts (v2.0.1) [26]. Differential expression analysis between treatment and control groups was performed using the DESeq2 package in R (v4.2.0) [27]. Genes were considered significantly differentially expressed if they met both of the following criteria: (1) |log_2_FC| > 1, corresponding to a minimum two‐fold change in expression; and (2) false discovery rate (FDR) < 0.05, as calculated using the Benjamini–Hochberg procedure for multiple testing correction. Only genes satisfying both thresholds were retained as significant DEGs for downstream analyses. The numbers of DEGs identified in each comparison are summarized in Table 2.

**TABLE 2:** Summary of differentially expressed genes (DEGs).

Cell type	Treatment versus Control	Total DEGs	Upregulated	Downregulated
HaCaT	PSO‐Cer versus Control	9152	4554	4598
HaCaT	Ceramide IIIB versus Control	10 860	5386	5474
HDF‐a	PSO‐Cer versus Control	6652	3366	3286
HDF‐a	Ceramide IIIB versus Control	4027	1923	2104

DEG criteria: |log_2_FC| > 1, FDR < 0.05 (DESeq2).

To interpret the biological functions of the identified DEGs, functional enrichment analysis was conducted using Gene Ontology (GO) [26] and Kyoto Encyclopedia of Genes and Genomes (KEGG) [25] via the clusterProfiler package (version 4.8.1) [25, 26]. Enrichment results with FDR < 0.05 were considered statistically significant. The analysis was conducted separately for all DEGs, as well as for up‐regulated and down‐regulated gene sets for each comparison. Finally, the results were visualized using the ggplot2 R package to generate dot plots and bar plots. Volcano plots displaying the distribution of DEGs are shown in Figure 6. In addition, the integrated transcriptomic heatmap is presented in Figure 7. KEGG pathway enrichment analysis results for HaCaT cells are shown in Figure 8 and for HDF‐a cells in Figure 9.

**FIGURE 6:**
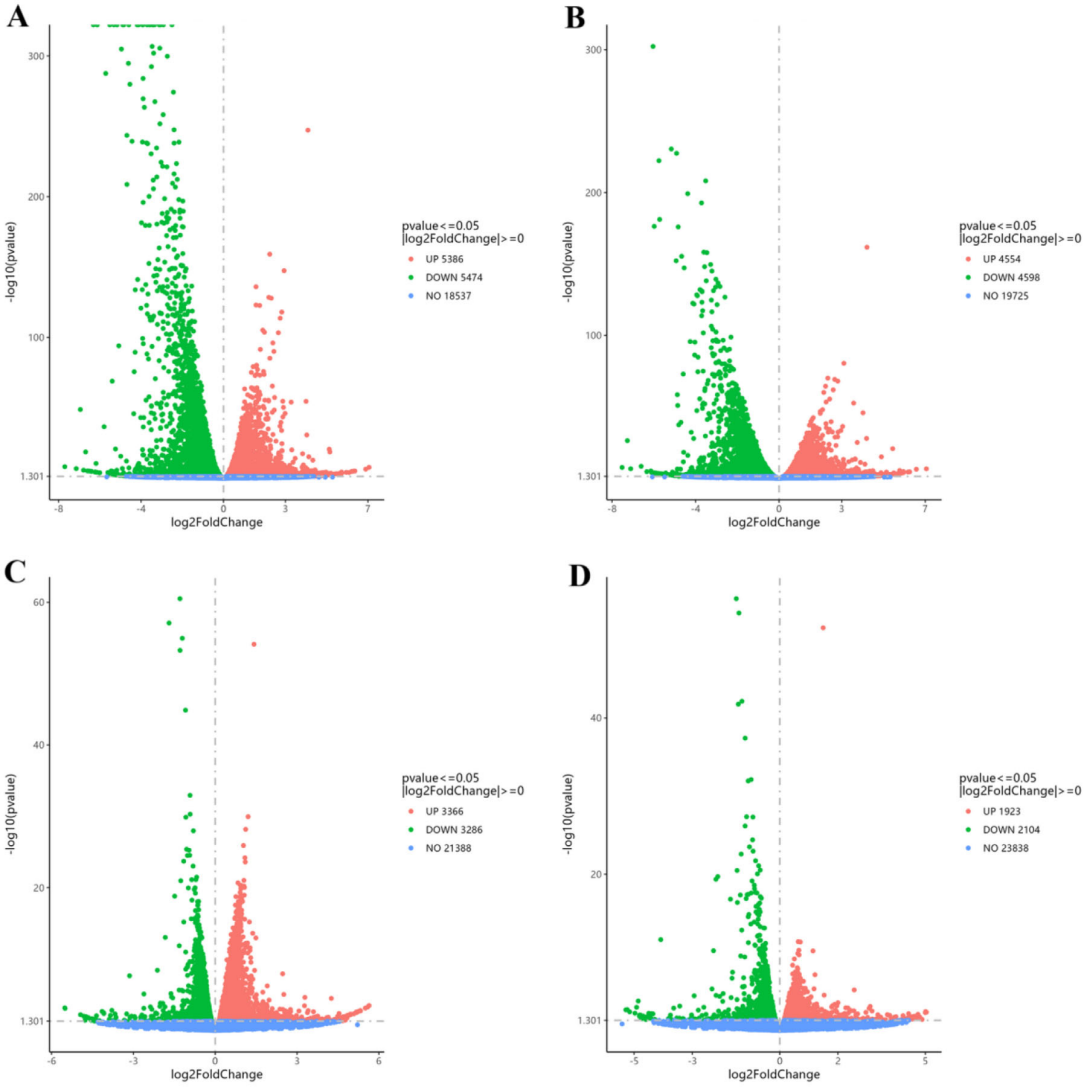
Volcano plots of differentially expressed genes (DEGs) in HaCaT and HDF‐a cells treated with PSO‐Cer and Ceramide IIIB. Volcano plots displaying the distribution of DEGs in (A) HaCaT cells treated with PSO‐Cer versus Control. (B) HaCaT cells treated with Ceramide IIIB versus Control. (C) HDF‐a cells treated with PSO‐Cer versus Control, and (D) HDF‐a cells treated with Ceramide IIIB versus Control. The *x*‐axis represents log_2_(Fold Change), and the *y*‐axis represents ‐log_10_(FDR). Red dots indicate significantly upregulated genes (|log_2_FC| > 1, FDR < 0.05), blue dots indicate significantly downregulated genes (|log_2_FC| < −1, FDR < 0.05), and gray dots indicate genes with no significant differential expression. The vertical dashed lines represent the |log_2_FC| = 1 threshold, and the horizontal dashed line represents the FDR = 0.05 significance threshold. DEG counts are summarized in Table 2.

**FIGURE 7:**
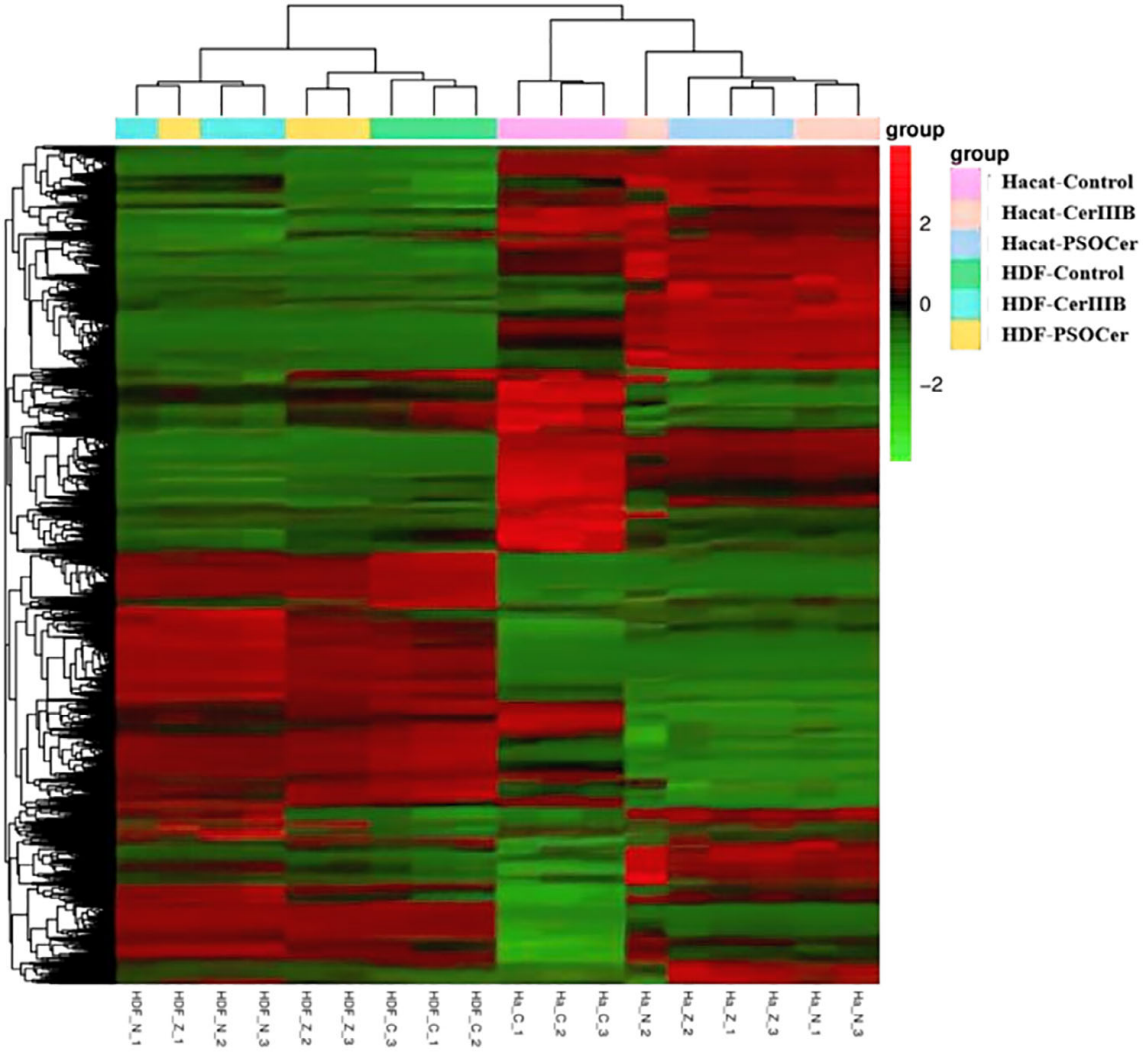
Integrated transcriptomic heatmap of differentially expressed genes in HaCaT keratinocytes and HDF‐a fibroblasts. The heatmap displays row‐wise Z‐score‐normalized expression values of the union of top‐ranked differentially expressed genes (|log_2_ fold change| > 1.0, FDR < 0.05) identified in HaCaT and HDF‐a cells following treatment with PSO‐Cer or Ceramide IIIB. Columns represent individual biological replicates arranged in fixed order by cell type and treatment group; no column clustering was applied to preserve group structure. Rows represent genes hierarchically clustered by Euclidean distance. The top annotation bars indicate cell type (HaCaT, HDF‐a) and treatment condition (Control, Ceramide IIIB, PSO‐Cer). The color scale indicates relative expression level (red: high expression; green: low expression). Complete DEG statistics are provided in Table 2.

**FIGURE 8:**
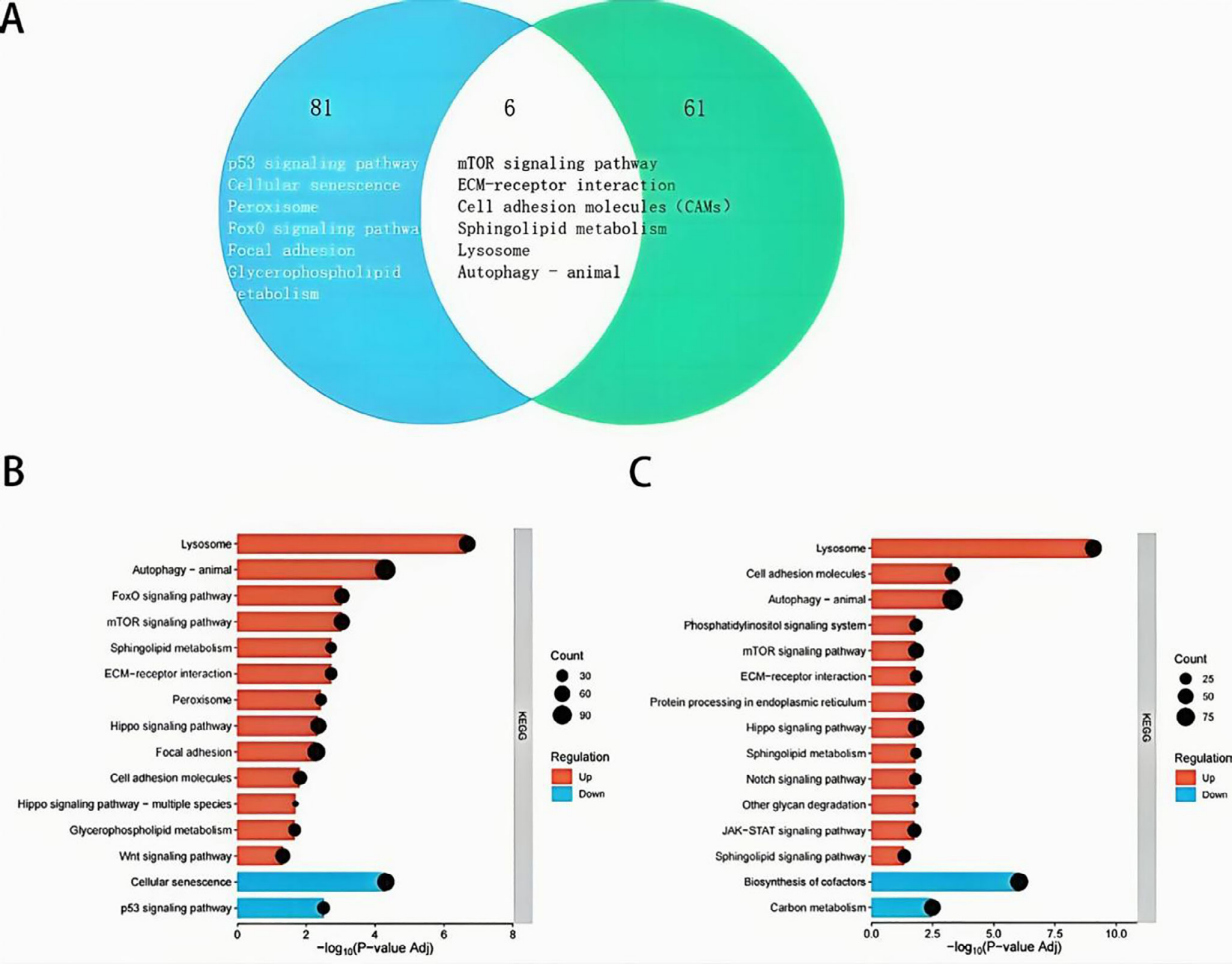
Transcriptomic profiling and KEGG pathway enrichment analysis of HaCaT cells following treatment with PSO‐Cer and Ceramide IIIB. (A) Venn diagram illustrating the number of differentially expressed genes (DEGs) in HaCaT cells treated with PSO‐Cer (left) or Ceramide IIIB (right) relative to the control group. (B,C) KEGG pathway enrichment analysis of DEGs in HaCaT cells treated with PSO‐Cer (B) or Ceramide IIIB (C). The top 20 significantly enriched pathways are presented, where dot size corresponds to the number of DEGs, and dot color indicates the false discovery rate (FDR), with red representing upregulated genes and blue representing downregulated genes.

**FIGURE 9:**
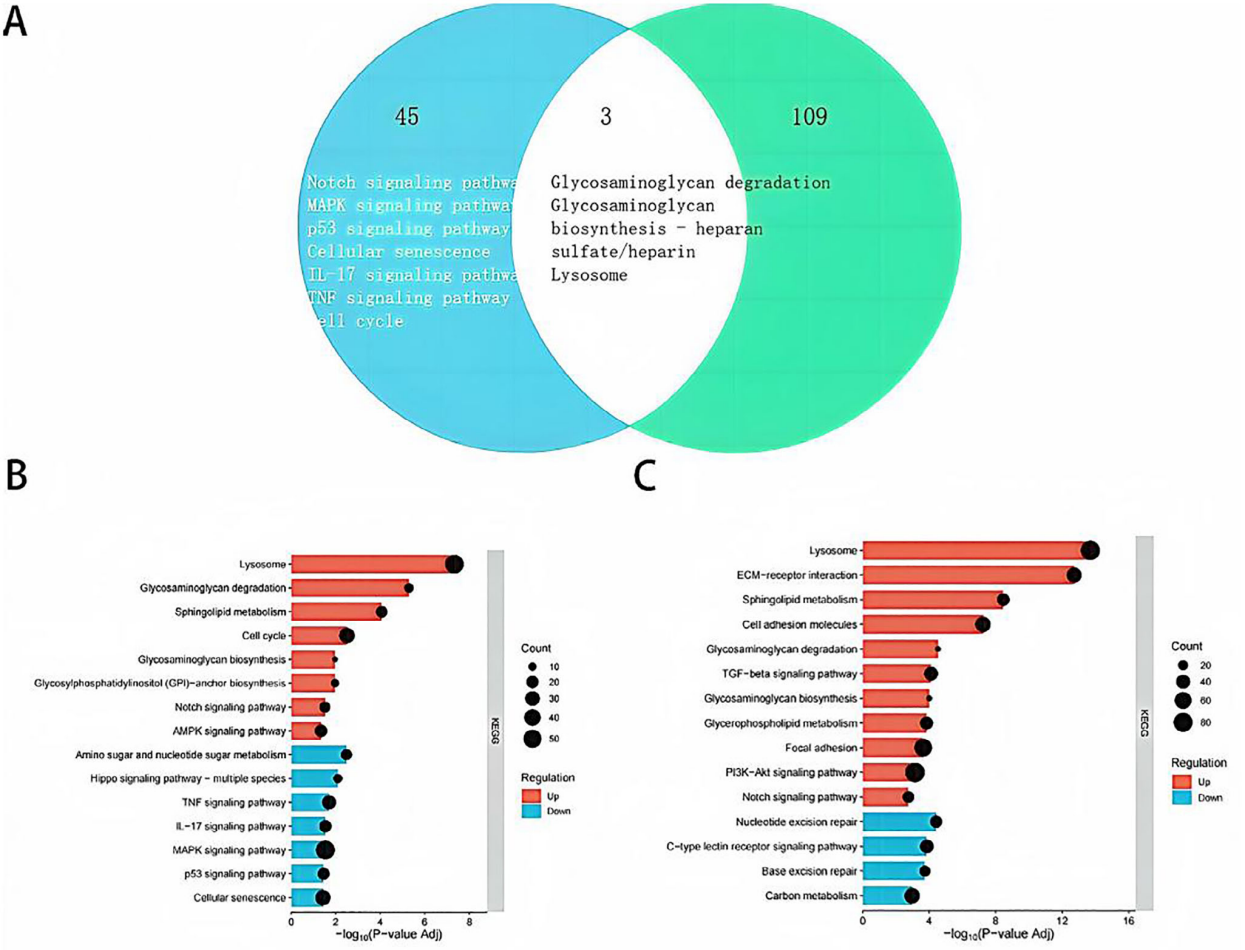
Transcriptomic profiling and KEGG pathway enrichment analysis of HDF‐a cells following treatment with PSO‐Cer and Ceramide IIIB. (A) Venn diagram illustrating the number of differentially expressed genes (DEGs) in HDF‐a cells treated with PSO‐Cer (left) or Ceramide IIIB (right) relative to the control group. (B,C) KEGG pathway enrichment analysis of DEGs in HDF‐a cells treated with PSO‐Cer (B) or Ceramide IIIB (C). The top 20 significantly enriched pathways are presented, where dot size corresponds to the number of DEGs, and dot color indicates the false discovery rate (FDR), with red representing upregulated genes and blue representing downregulated genes.

### 2.7 Statistical Analysis

All experiments, except for mRNA‐seq, were independently repeated at least three times. The final data are presented as the mean ± standard deviation (mean ± SD). Raw counts from the mRNA‐seq analysis were normalized using the Tags Per Million (TPM) method. Statistical comparisons were performed using Student's *t*‐test (for two groups) and one‐way ANOVA (≥ three groups). A *p*‐value of < 0.05 (*), < 0.01 (**), and < 0.001 (***) was considered statistically significant. In addition, all graphs were generated using GraphPad Prism 8 (GraphPad Software Inc., USA).

## 3 Results

### 3.1 Composition of PSO‐Cer

The composition of the enzymatically synthesized PSO‐Cer was characterized with HPLC‐CAD (Figure 2). The integral results of the chromatographic peaks, including all detected peaks and their assignments, are provided in Table S2, with the major ceramide species identified based on their retention times and the characteristic fatty acid profile of the precursor PSO [28]. The HPLC analysis demonstrated that PSO‐Cer was a mixture of several ceramide species, predominantly composed of ceramides bearing C18:3 (α‐linolenic acid), C18:1 (oleic acid), C18:2 (linoleic acid), C16:0 (palmitic acid), and C18:0 (stearic acid) acyl chains. Together, these five major species constituted over 95% of the total detected composition. Notably, the ceramide with an α‐linolenic acyl chain (C18:3) was the most abundant single component, accounting for 34.49% of the relative peak area.

To further identify the specific molecular species, PSO‐Cer was subjected to LC‐QTOF analysis (Table 1). The high‐resolution mass spectrometry confirmed the presence of major ceramide species, with the most abundant precursor ions corresponding to [M+H]^+^ adducts of phytoceramides containing C18:3 (α‐linolenic, *m/z* 578.5119), C18:2 (linoleic, *m/z* 580.5290), C18:1 (oleic, *m/z* 582.5438), and C16:0 (palmitic, *m/z* 556.5270) acyl chains. CID fragmentation of these precursor ions yielded characteristic fragment ions including [M+H‐H_2_O]^+^ (neutral loss of water) and the diagnostic phytosphingosine backbone ion at *m/z* 282.2796 [t18:0−2H_2_O+H]^+^ (C_18_H_36_NO^+^, calc. 282.2792, error 1.8 ppm), confirming the phytosphingosine moiety. For the most abundant C18:3 species, an additional fragment ion at *m/z* 261.2216 corresponding to the C18:3 fatty acyl fragment [C_17_H_29_O]^+^ was also observed, further confirming the α‐linolenic acyl chain. C18:0 (stearic) ceramide was not detected by LC‐QTOF, likely due to its lower abundance (9.46% by HPLC‐CAD) and/or reduced ionization efficiency of saturated species. This analysis provided unequivocal identification of the synthesized ceramide species, corroborating the HPLC‐CAD results and validating the specificity of the enzymatic synthesis. The complete LC‐QTOF identification data, including all detected adduct ions and fragment species, are provided in Table S1.

### 3.2 Cytotoxicity Assessment and Concentration Selection

The CCK‐8 assay was employed to evaluate the cytotoxicity of two ceramides, Ceramide IIIB and PSO‐Cer, on HaCaT and HDF‐a cell lines across a concentration gradient from 0.1 to 50 µg/mL (Figure 3). In HaCaT cells (Figure 3A), both compounds exhibited no significant cytotoxicity at concentrations ranging from 0.1 to 25 µg/mL, with cell viability maintained between approximately 95% and 115%. However, a significant cytotoxic effect was observed at 50 µg/mL, leading to a pronounced reduction in viability (*p* < 0.001). In contrast, HDF‐a cells (Figure 3B) demonstrated a distinct response, where treatments from 0.1 to 10 µg/mL with ceramide resulted in cell viability levels between 106% and 123%, potentially indicating enhanced metabolic activity, while a return to baseline viability occurred at 25 µg/mL. Moreover, the marked cytotoxicity was subsequently evident at the 50 µg/mL concentration, reducing viability to approximately 65%, an effect that was notably less severe than that observed in HaCaT cells.

In summary, both ceramides exhibited no significant cytotoxicity against either HaCaT or HDF‐a cells within the broad concentration range of 0.1–25 µg/mL. A clear cytotoxic threshold was identified at 50 µg/mL for both compounds. Thus, the concentrations of 1 and 10 µg/mL were selected for subsequent qPCR and Western blot analyses because both concentrations reside within the unequivocally non‐cytotoxic range for all tested cell lines and compounds, with 1 µg/mL as the low dose and 10 µg/mL as the high dose to evaluate dose‐dependent efficacy.

### 3.3 Effects on Barrier‐Related Gene Expression (qPCR)

Compared to the untreated control, the mRNA expression levels of key genes involved in keratinocyte terminal differentiation and natural moisturizing factor (NMF) formation, including filaggrin (FLG), Caspase‐14 (CASP14), and bleomycin hydrolase (BLMH), were significantly upregulated in HaCaT cells following treatment with both PSO‐Cer and Ceramide IIIB at concentrations of 1 and 10 µg/mL (Figure 4A–C). For FLG (Figure 4A), a consistent and dose‐dependent increase in expression was observed for both compounds, with the 10 µg/mL concentration yielding the highest levels. A more dramatic upregulation was evident for CASP14 (Figure 4B) and BLMH (Figure 4C), with both genes showing a strong dose‐responsive induction. The 10 µg/mL treatment of Ceramide IIIB resulted in a substantial stimulation of CASP14 expression, exceeding a 2.2‐fold increase on average.

The mRNA expression levels of two critical genes involved in cornified envelope formation, Involucrin (IVL) and transglutaminase‐1 (TGM1), were significantly upregulated in HaCaT cells after treatment with PSO‐Cer and Ceramide IIIB at 1 and 10 µg/mL (Figure 4D,E). For IVL (Figure 4D), a marked and consistent increase in expression was observed for both compounds. A similar upregulatory trend was evident for TGM1 (Figure 4E), with PSO‐Cer at 10 µg/mL demonstrating the highest efficacy in stimulating TGM1 transcription. The mRNA expression of ultra‐long‐chain ceramide synthases, ceramide synthase 3 (CERS3) and elongation of very long chain fatty acids protein 4 (ELOVL4), was significantly modulated in HaCaT cells following treatment with PSO‐Cer and Ceramide IIIB (Figure 4F,G). Both ceramides increased CERS3 expression (Figure 4F), with PSO‐Cer demonstrating a marginally stronger effect at both concentrations. Both compounds also potently stimulated ELOVL4 expression (Figure 4G) in a clear dose‐dependent manner, with the 10 µg/mL concentration inducing the highest mRNA levels. The magnitude of induction was substantially greater for ELOVL4 than for CERS3, suggesting that the primary transcriptional response to exogenous ceramide treatment may target the fatty acid elongation step.

### 3.4 Effects on ECM Gene Expression in UVB Model (qPCR)

The mRNA expression levels of key ECM components, COL1A1, COL3A1, and ELN were quantitatively assessed in a UVB‐induced cellular senescence model of human dermal fibroblasts (HDF‐a). As illustrated in Figure 4H–J, UVB irradiation (the model group) significantly downregulated the expression of all three genes compared to the non‐irradiated Control (*p* < 0.01). Meanwhile, a restorative effect on gene expression was demonstrated after treatment with both PSO‐Cer and Ceramide IIIB at 1 and 10 µg/mL. For COL1A1 (Figure 4H), the 10 µg/mL concentration of both ceramides restored mRNA levels to near or even slightly above the Control level (*p* < 0.01). A similar trend was observed for COL3A1 (Figure 4I) and ELN (Figure 4J). The 10 µg/mL concentration consistently produced a more pronounced restorative effect across all three genes for both compounds, often normalizing expression to baseline control levels.

### 3.5 Western Blot Validation of Protein Expression

To determine whether the observed transcriptional changes were translated into functional protein expression, Western blot analysis was performed for all eight target proteins (FLG, CASP14, IVL, TGM1, ELOVL4, CERS3, COL1A1, and ELN). The representative Western blot bands are shown in Figure 5.

In HaCaT cells (Figure 5A–F and Table 2), both PSO‐Cer and Ceramide IIIB significantly increased protein expression of all barrier‐related targets in a dose‐dependent manner. FLG protein increased from 0.81 (Control) to 0.98 at 10 µg/mL PSO‐Cer and 1.19 at 10 µg/mL ceramide IIIB (*p* < 0.001 for both). CASP14 protein showed the most robust induction, increasing from 0.54 (Control) to 1.38 at 10 µg/mL PSO‐Cer and 1.42 at 10 µg/mL Ceramide IIIB (*p* < 0.001). IVL, TGM1, ELOVL4, and CERS3 all showed consistent dose‐dependent increases, corroborating the qPCR findings.

In the UVB‐induced HDF‐a photoaging model (Figure 5G,H and Table 2), UVB irradiation significantly reduced COL1A1 protein from 1.04 (Control) to 0.43 (Model, *p* < 0.001) and ELN protein from 0.87 to 0.46 (*p* < 0.001). Treatment with both ceramides effectively reversed this downregulation. COL1A1 was restored to 1.05 at 10 µg/mL PSO‐Cer and 1.30 at 10 µg/mL Ceramide IIIB (*p* < 0.001 vs. Model). ELN was restored to 0.90 at 10 µg/mL PSO‐Cer and 0.86 at 10 µg/mL Ceramide IIIB (*p* < 0.001 vs. Model). These results confirm that the transcriptional changes observed by qPCR translate into functional protein expression. Complete densitometric data are provided in Table S3A,B. In addition, a summary of qPCR‐western blot consistency is presented in Table 3.

**TABLE 3:** Consistency between qPCR and Western blot results.

Target	Method	Control	PSO‐Cer 1 µg	PSO‐Cer 10 µg	Cer IIIB 1 µg	Cer IIIB 10 µg	Consistency
FLG	qPCR	1.00	1.09	1.21	1.23	1.47	Yes
FLG	WB	0.81	0.88	0.98	1.00	1.19	Yes
CASP14	qPCR	1.00	2.00	2.56	1.96	2.63	Yes
CASP14	WB	0.54	1.08	1.38	1.06	1.42	Yes
IVL	qPCR	1.00	1.05	1.17	1.23	1.26	Yes
IVL	WB	0.86	0.90	1.01	1.06	1.08	Yes
TGM1	qPCR	1.00	1.30	1.52	1.18	1.95	Yes
TGM1	WB	0.56	0.73	0.85	0.66	1.09	Yes
ELOVL4	qPCR	1.00	2.37	2.26	3.19	3.37	Yes
ELOVL4	WB	0.27	0.64	0.61	0.86	0.91	Yes
CERS3	qPCR	1.00	1.28	1.25	1.62	1.58	Yes
CERS3	WB	0.57	0.72	0.70	0.92	0.89	Yes
COL1A1	qPCR	1.00	2.40	2.44	2.33	3.02	Yes
COL1A1	WB	1.04	1.03	1.05	1.00	1.30	Yes
ELN	qPCR	1.00	1.65	1.96	1.85	1.87	Yes
ELN	WB	0.87	0.76	0.90	0.85	0.86	Yes

### 3.6 Transcriptomic Profiling and Pathway Enrichment Analysis

Differential expression analysis revealed cell type‐specific transcriptional responses to the two ceramides. As summarized in Table 1, in HaCaT cells, Ceramide IIIB induced 10 860 DEGs (5386 upregulated, 5474 downregulated), while PSO‐Cer induced 9152 DEGs (4554 upregulated, 4598 downregulated). In HDF‐a cells, PSO‐Cer induced 6,652 DEGs (3366 upregulated, 3286 downregulated), compared to 4027 DEGs (1923 upregulated, 2104 downregulated) for Ceramide IIIB. Volcano plots (Figure 6A–D) and heatmaps (Figure 7) were generated to visualize the distribution and clustering of DEGs across treatment groups.

The transcriptomic profiles of HaCaT keratinocytes treated with PSO‐Cer and Ceramide IIIB revealed significant modulations in key signaling pathways related to skin barrier function, cellular metabolism, and stress response. Both ceramides demonstrated a remarkable convergence in upregulating pathways central to barrier repair and epidermal differentiation, including the mTOR signaling pathway, ECM–receptor interaction, cell adhesion molecules (CAMs), sphingolipid metabolism, lysosomes, and autophagy (Figure 8B,C). Notably, PSO‐Cer exhibited a distinct and broader spectrum of bioactivity (Figure 8B). It specifically upregulated pathways associated with antioxidant defense (Peroxisome, FoxO signaling) and cellular remodeling (Focal adhesion, Glycerophospholipid metabolism), while concurrently downregulating key drivers of cellular aging (p53 signaling pathway, Cellular senescence). This distinct regulation suggested that PSO‐Cer possesses multifaceted efficacy beyond basic barrier repair.

The differential regulation observed with PSO‐Cer points to its distinct functionality (Figure 4B,C). The upregulation of Peroxisome and FoxO signaling pathways provided a robust defense mechanism against oxidative stress by enhancing the breakdown of very‐long‐chain fatty acids (VLCFAs) and reactive oxygen species (ROS), thereby protecting the integrity of barrier lipids and cellular structures. More importantly, the significant downregulation of the p53 signaling and Cellular senescence pathways suggested a promising anti‐aging potential. By mitigating the DNA damage response and breaking the cycle that led to irreversible cell cycle arrest, PSO‐Cer has helped maintain the proliferative capacity and longevity of keratinocytes. Furthermore, the specific upregulation of Focal adhesion and Glycerophospholipid metabolism underscored its role in promoting cellular migration (crucial for wound healing) and optimizing the composition and fluidity of cell membranes, which was vital for forming effective tight junctions.

In human dermal fibroblasts (HDF‐a), both PSO‐Cer and Ceramide IIIB demonstrated marked efficacy in promoting ECM remodeling and imparting anti‐aging benefits (Figure 9B,C). The most significant effects were observed in the upregulation of Glycosaminoglycan (GAG) metabolism (both degradation and biosynthesis pathways), Lysosome activity, the Notch signaling pathway, and the Cell cycle. Concurrently, both ceramides effectively downregulated key drivers of inflammation and cellular senescence, specifically the IL‐17 signaling pathway, TNF signaling pathway, p53 signaling pathway, and the Cellular senescence pathway. The MAPK signaling pathway was also suppressed, indicating a potential mechanism for reducing ECM degradation. The observed downregulations of the p53 and Cellular senescence pathways were critical. By mitigating the DNA damage response and cellular stress pathways that triggered irreversible growth arrest, both ceramides helped in preserving the replicative capacity of dermal fibroblasts, which was essential for long‐term skin vitality and repair (Figure 5B,C). The simultaneous suppression of the IL‐17 and TNF signaling pathways directly counteracted the chronic, low‐grade inflammation (“inflammaging”) that was a hallmark of aged skin, reducing the inflammatory drive that degrades collagen and elastin. In addition, the activation of the cell cycle indicated that these ceramides actively promote fibroblast proliferation, accelerating the repopulation of the dermis with functional, matrix‐producing cells. Furthermore, enhanced Lysosomal activity guaranteed efficient clearance of damaged cellular components and oxidized proteins, maintaining cellular homeostasis and supporting the overall anabolic (ECM‐building) environment.

## 4 Discussion

The ceramide species profile of PSO‐Cer closely mirrored the characteristic fatty acid composition of its botanical source PSO. Literature consistently reported that PSO was notably rich in α‐linolenic acid (C18:3), which constituted >60% of its total fatty acids, followed by oleic acid (C18:1, >12%), linoleic acid (C18:2, >13%), saturated fatty acids like palmitic (C16:0, ∼7%) and stearic acid (C18:0, ∼1.4%) [28]. The high abundance of C18:3‐ceramide (34.49%) in PSO‐Cer directly reflected this distinctive fatty acid signature. The presence of a high proportion of polyunsaturated ceramides in PSO‐Cer may confer distinct biophysical properties and enhanced fluidity to the skin's lipid lamellae, potentially leading to effective moisturizing efficacy and skin barrier benefits compared to ceramides derived from sources with lower degrees of unsaturation, such as Ceramide IIIB (primarily oleic acid). Probably, this distinct composition formed the basis for investigating its differentiated biological activity in subsequent cellular assays.

Additionally, the coordinated upregulation of FLG, CASP14, and BLMH was critically significant for skin barrier function. FLG was a key structural protein in the cornified envelope, while its proteolytic processing by CASP14 and BLMH into free amino acids was essential for NMF generation, which was responsible for skin hydration [9]. The ability of both ceramides to enhance this entire functional module indicated a comprehensive role in promoting epidermal differentiation and improving the skin's natural moisturizing capacity. The pronounced upregulation of CASP14 was particularly compelling, as this enzyme was crucial for the specific hydrolysis of filaggrin and was a marker for terminal keratinocyte differentiation. In particular, the potent effect of PSO‐Cer suggested a strong activation of this final step in NMF biogenesis. The robust efficacy of PSO‐Cer, despite its different fatty acid profile, underscored its potential as a high‐value plant‐derived alternative. By stimulating the expression of these fundamental barrier genes, both ceramides demonstrated a mechanism of action that goes beyond passive lipid supplementation to actively regulate keratinocyte biology, thereby strengthening the epidermal barrier and enhancing skin hydration. This provided a solid molecular basis for their use in improving conditions associated with barrier dysfunction, such as dry skin and atopic dermatitis.

Moreover, the coordinated induction of IVL and TGM1 was of paramount importance for skin barrier integrity. IVL served as a crucial structural scaffold protein for the cornified envelope, while TGM1 was the key enzyme that cross‐links IVL and other substrates to form this insoluble, protective structure at the cell periphery [9]. The ability of both PSO‐Cer and Ceramide IIIB to upregulate this “scaffold‐and‐crosslink” module demonstrated a potent role in promoting the terminal differentiation of keratinocytes and fortifying the skin's primary barrier. This enhanced gene expression likely translated to a more resilient and functionally competent stratum corneum. The robust efficacy of PSO‐Cer was particularly noteworthy, as it positions this plant‐derived ceramide as a viable and potent alternative to traditional ceramides for improving skin barrier function. By stimulating the expression of these fundamental structural genes, both ceramides demonstrated a mechanism that actively supports the formation of a robust physical barrier, which was essential for protecting against environmental insults and preventing transepidermal water loss. This provided a strong molecular rationale for their use in skincare formulations aimed at barrier repair and conditions like xerosis or atopic dermatitis.

Besides, the coordinated upregulation of CERS3 and ELOVL4 was critically significant for skin barrier homeostasis. Recent reviews have highlighted the importance of phytosphingosine‐based ceramides in maintaining skin barrier homeostasis and their therapeutic potential for atopic skin conditions [29]. ELOVL4 was essential for producing the ultra‐long‐chain (C28‐C36) fatty acids that were characteristic and functionally vital for stratum corneum ceramides, while CERS3 utilized these fatty acids to synthesize the specific ceramide species crucial for forming impermeable lipid lamellae [5]. The potent induction of ELOVL4, in particular, indicated that both PSO‐Cer and Ceramide IIIB were not merely passive lipid supplements but active signaling molecules that can enhance the skin's intrinsic capacity to produce its own optimal, long‐chain ceramides. This represented a powerful “feed‐forward” mechanism, where the application of specific ceramides stimulated the endogenous synthesis of barrier‐competent lipids, thereby promoting a more resilient and functional skin barrier. The efficacy of PSO‐Cer in this regard underscored its potential as a high‐value, plant‐derived bioactive for therapeutic barrier restoration.

The downregulation of COL1A1, COL3A1, and ELN following UVB exposure was a canonical feature of skin photoaging, leading to the loss of skin strength, elasticity, and structural integrity. The ability of both PSO‐Cer and Ceramide IIIB to mitigate this downregulation was highly significant. It suggested that these ceramides do not merely provide passive structural support but actively participate in cellular defense and ECM homeostasis, potentially by modulating signaling pathways involved in the UVB stress response and collagen synthesis, such as the transforming growth factor‐beta (TGF‐β) or mitogen‐activated protein kinase (MAPK) pathways. The efficacy at 10 µg/mL underscored the importance of selecting a biologically active, non‐cytotoxic concentration for functional studies, as validated by our initial cytotoxicity screening. Notably, the comparable performance of PSO‐Cer to the well‐established Ceramide IIIB was particularly promising. Given that PSO‐Cer was enriched with polyunsaturated fatty acid chains like α‐linolenic acid, its protective effect may be augmented by inherent anti‐inflammatory or antioxidant properties, which could help mitigate the oxidative stress component of UVB damage. This positioned PSO‐Cer not just as a structural lipid replacement but as a multifunctional active ingredient with the potential to combat key molecular events in skin aging by promoting the synthesis of essential dermal components.

Particularly, in gene transcriptome sequencing, the findings revealed both PSO‐Cer and Ceramide IIIB as highly effective activators of skin barrier reconstruction in keratinocytes. Their shared ability to co‐activate mTOR‐driven differentiation, sphingolipid synthesis, and autophagic clearance presented a powerful, multi‐pronged strategy for repairing compromised skin barriers, making them highly relevant for conditions like atopic dermatitis and xerosis. However, the distinct pharmacological profile of PSO‐Cer elevated it from a mere barrier repair agent to a comprehensive protective and anti‐aging active. Its capacity to simultaneously enhance barrier function while activating antioxidant (Peroxisome, FoxO) and suppressing pro‐aging pathways (p53, Senescence) aligned with the concept of “geroprotection” in skin cells. This was particularly significant in the context of extrinsic skin aging, where UV radiation and environmental pollutants incessantly generate oxidative stress and DNA damage, accelerating the senescence of epidermal cells and impairing barrier function. The distinct fatty acid profile of PSO‐Cer, particularly its high content of α‐linolenic acid, likely contributed to this enhanced bioactivity. Polyunsaturated fatty acids are known precursors to anti‐inflammatory mediators and can influence membrane fluidity and signal transduction. This may explain the more nuanced regulation of stress‐response and metabolic pathways observed with PSO‐Cer compared to the more standardized Ceramide IIIB.

In conclusion, while both ceramides effectively enhanced the skin barrier, PSO‐Cer transcends its role as a mere barrier repair agent. Its distinct capacity to integrate barrier strengthening with intrinsic antioxidant and anti‐senescence mechanisms positions it as a promising candidate for advanced skincare formulations. These formulations can be aimed not only at correcting barrier dysfunction but also at preventing premature skin aging and promoting long‐term epidermal health. Additionally, the transcriptomic data from HDF‐a cells revealed that PSO‐Cer and Ceramide IIIB functioned as modulators of dermal gene expression, orchestrating a shift from a catabolic, pro‐inflammatory state to an anabolic, rejuvenating state. In particular, their impact on GAG metabolism was noteworthy. Rather than a blunt stimulation, the fine‐tuned regulation of both synthesis and degradation pathways suggested an intelligent remodeling of the dermal matrix to optimize its hydrating and signaling properties. The dual action of simultaneously boosting collagen production (via Notch) and protecting it from degradation (via MAPK downregulation and inflammation suppression) provided a powerful strategy to combat the net collagen loss that characterizes photoaged and chronologically aged skin. This was further reinforced by their geroprotective potential, delaying fibroblast senescence by inhibiting the p53 pathway and clearing potential senescent cells via activated autophagy. By preventing fibroblasts from entering a senescent, pro‐inflammatory state (SASP), these ceramides helped break a vicious cycle that perpetuates tissue degeneration. Moreover, in the dermal compartment, both ceramides demonstrated a comprehensive, multi‐target mechanism of action. They not only stimulated the synthesis of essential structural components (collagen, GAGs) but also proactively preserved the cellular machinery responsible for their production by mitigating key drivers of aging, inflammation, ECM degradation, and cellular senescence. This positioned them, and particularly PSO‐Cer with its previously noted antioxidant advantages, as highly effective actives for anti‐aging and skin‐rejuvenating applications aimed at improving dermal density, hydration, and anti‐aging. Compared to previously reported plant‐derived ceramides, PSO‐Cer exhibits several distinctive features. Choi et al. [14] reported that ceramides derived from Korean traditional plant oils (*C. japonica*, *P. mume*, and *S. indicum*) enhanced skin barrier functions and stimulated epidermal gene expression. However, these ceramides were primarily composed of saturated or mono‐unsaturated fatty acyl chains (predominantly C16:0 and C18:1). In contrast, PSO‐Cer is notably enriched in polyunsaturated C18:3 (α‐linolenic acid, 34.49%), which may confer additional antioxidant and anti‐inflammatory benefits through its highly unsaturated structure. Oh et al. [16] demonstrated that novel phytoceramides containing fatty acids of diverse chain lengths improved stratum corneum properties, but their ceramide mixtures did not achieve the level of polyunsaturation observed in PSO‐Cer. The high α‐linolenic acid content of PSO‐Cer positions it as a distinct plant‐derived ceramide with potential for enhanced bioactivity through combined barrier repair and antioxidant mechanisms.

Several limitations of this study should be acknowledged. First, the biological activity of PSO‐Cer was evaluated exclusively in vitro using immortalized cell lines (HaCaT and HDF‐a); in vivo validation in animal models or human skin equivalents would be required to confirm translational potential. Second, while transcriptomic and protein‐level changes were demonstrated, functional readouts such as transepithelial electrical resistance (TER), permeability assays, or collagen deposition were not performed. Third, the comparison was limited to Ceramide IIIB as a single comparator; future studies should include a broader panel of commercially relevant ceramides. Fourth, while LC‐QTOF analysis provided high‐resolution mass confirmation, full nuclear magnetic resonance (NMR) characterization (^1^H, ^1^ ^3^C, 2D NMR) would further strengthen structural confirmation. Fifth, atomic force microscopy (AFM) studies of PSO‐Cer‐containing lipid monolayers could provide valuable biophysical insights into membrane organization and packing behavior. Sixth, the conclusions regarding anti‐aging and barrier repair effects are based on gene and protein expression changes measured at a single time point (24 h); time‐course analyses would be valuable for future investigation.

## 5 Conclusion

In summary, this study successfully established an enzymatic route for synthesizing a novel plant‐based ceramide, PSO‐Cer, which faithfully mirrors the distinct fatty acid profile of its PSO origin. The comprehensive in vitro evaluation confirms that PSO‐Cer was not only a safe and effective alternative to Ceramide IIIB but also possessed an effective, multifaceted bioactivity profile. Both ceramides potently stimulated skin barrier repair in keratinocytes and protected fibroblasts from UVB‐induced ECM damage. The transcriptomic analysis revealed the pivotal distinction: PSO‐Cer modulated a broader signaling network. It concurrently activated antioxidant defenses (Peroxisome, FoxO) and suppressed core aging pathways (p53, Cellular Senescence) in keratinocytes, while fine‐tuning glycosaminoglycan metabolism and suppressing inflammation in fibroblasts. Consequently, PSO‐Cer extended beyond the conventional role of a barrier‐lipid supplement, emerging as a multifunctional active that simultaneously addresses barrier dysfunction, oxidative stress, chronic inflammation, and cellular senescence. Its high α‐linolenic acid content likely underlied these enhanced benefits, positioning PSO‐Cer as a sustainable and efficacious plant‐derived ingredient for innovative anti‐aging and barrier‐repair formulations.

## Author Contributions

**Jumao Yuan**: conceptualization, methodology, formal analysis, writing – original draft, supervision, project administration, funding acquisition. **Ning Hao**: methodology, validation, investigation, data curation. **Borui Shi**: data curation and formal analysis. **Zhenzhen Wang**: methodology, validation, investigation, data curation. **Jie Yu**: resources, writing – review and editing. **Nuo Shi**: visualization, software, formal analysis. **Lin Lin**: conceptualization, supervision, writing – review and editing.

## Funding

This study was supported by the National Key Research and Development Program of China (2022YFC2104800). The funding bodies provided financial support for the research activities, but the multifunctional active for advanced skincare formulations aimed at comprehensive skin health was not involved in the study design, data collection, analysis, interpretation, or manuscript writing.

## Conflicts of Interest

The authors declare no conflicts of interest.

## Declaration of Generative AI in Scientific Writing

During the preparation of this manuscript, artificial intelligence tools, ChatGPT (OpenAI GPT‐4, version GPT‐4‐0613), were used solely to enhance language clarity and readability. No AI tools were used for data generation, data analysis, image processing, experimental design, or the formation of scientific conclusions.

## Supporting information




**Supporting File 1**: cbdv71808‐sup‐0001‐SuppMat.docx

## Data Availability

The data that support the findings of this study are available in the Supporting Information of this article.
