# Supplementary material for: Enzymatic Synthesis of a Novel α‐Linolenic Acid‐Enriched Phytoceramide From Perilla Seed Oil and Its Multifunctional Bioactivity in Skin Barrier Repair and Anti‐Aging
Source: Chem Biodivers. 2026 Oct 11;23(10):e71808. doi: 10.1002/cbdv.71808 (PMC13655634; doi:10.1002/cbdv.71808)
Supplement: Supplementary file 1 [file CBDV-23-e71808-s001.docx]

**Tables**

**Table S1. Complete LC-QTOF identification data of PSO-Cer.**

| No. | Component group | Proposed species | Adduct | Obs. m/z | RT (min) | Error (ppm) | Rel. intensity (%) |
| --- | --- | --- | --- | --- | --- | --- | --- |
| 1 | 578.5119 / 30.96 | Cer(t18:0/18:3) | [M+H]⁺ | 578.5119 | 30.96 | −5.0 | 100.0 |
| 2 | 578.5119 / 30.96 | Cer(t18:0/18:3) | [M+Na]⁺ | 600.4945 | 41.71 | −3.8 | 15.2 |
| 3 | 580.5290 / 41.57 | Cer(t18:0/18:2) | [M+H]⁺ | 580.5290 | 41.57 | −2.4 | 10.4 |
| 4 | 580.5290 / 41.57 | Cer(t18:0/18:2) | [M+Na]⁺ | 602.5094 | 41.78 | −5.0 | 7.1 |
| 5 | 582.5438 / 41.76 | Cer(t18:0/18:1) | [M+H]⁺ | 582.5438 | 41.76 | −3.9 | 14.0 |
| 6 | 582.5438 / 41.76 | Cer(t18:0/18:1) | [M+Na]⁺ | 604.5249 | 41.70 | −5.3 | 8.3 |
| 7 | 556.5270 / 36.55 | Cer(t18:0/16:0) | [M+H]⁺ | 556.5270 | 36.55 | −6.1 | 2.0 |
| **8** | **282.2796 / 21.94** | **Phytosphingosine backbone** | **[t18:0−2H₂O+H]⁺** | **282.2796** | **21.94** | **1.8** | **21.0** |
| **9** | **261.2216 / 23.52** | **C18:3 fatty acyl fragment** | **[C₁₇H₂₉O]⁺** | **261.2216** | **23.52** | **−1.5** | **2.1** |
| **10** | **279.2319 / 25.47** | **Linoleic acid (free)** | **[M-H]⁻** | **279.2319** | **25.47** | **−1.8** | **—** |
| **11** | **277.2172 / 24.49** | **γ-Linolenic acid (free)** | **[M-H]⁻** | **277.2172** | **24.49** | **1.4** | **—** |

**Table S2. PSO-Cer composition by HPLC-CAD analysis (full integration data).**

| Peak No. | RT (min) | Peak area (pA·min) | Peak height (pA) | Rel. area (%) | Assigned species | Identification basis |
| --- | --- | --- | --- | --- | --- | --- |
| 1 | 15.264 | 0.533 | 4.421 | 0.60 | — | — |
| 2 | 18.802 | 0.711 | 4.120 | 0.80 | — | — |
| 3 | 20.295 | 30.525 | 156.268 | 34.49 | C18:3 ceramide | LC-QTOF [M+H]⁺ |
| 4 | 22.506 | 15.269 | 64.288 | 17.25 | C18:2 ceramide | LC-QTOF [M+H]⁺ |
| 5 | 24.713 | 11.045 | 38.698 | 12.48 | C16:0 ceramide | LC-QTOF [M+H]⁺ |
| 6 | 25.710 | 19.379 | 67.743 | 21.89 | C18:1 ceramide | LC-QTOF [M+H]⁺ |
| 7 | 27.542 | 1.058 | 3.956 | 1.20 | — | — |
| 8 | 30.761 | 8.370 | 25.468 | 9.46 | C18:0 ceramide | RT correlation |
| 9 | 32.195 | 0.865 | 2.888 | 0.98 | — | — |
| 10 | 39.656 | 0.759 | 1.535 | 0.86 | — | — |

**Table S3A. Raw densitometric data for Western blot analysis (HaCaT cells).**

| Target | Group | Target IDV | ACTIN IDV | Ratio (Target/ACTIN) |
| --- | --- | --- | --- | --- |
| FLG | Control | 243792 | 301506 | 0.81 |
|  | PSO-Cer 1 μg/mL | 271603 | 309347 | 0.88 |
|  | PSO-Cer 10 μg/mL | 305233 | 312794 | 0.98 |
|  | Cer IIIB 1 μg/mL | 314960 | 314357 | 1.00 |
|  | Cer IIIB 10 μg/mL | 383245 | 320720 | 1.19 |
| CASP14 | Control | 238054 | 438197 | 0.54 |
|  | PSO-Cer 1 μg/mL | 476079 | 441280 | 1.08 |
|  | PSO-Cer 10 μg/mL | 606183 | 440274 | 1.38 |
|  | Cer IIIB 1 μg/mL | 472181 | 445143 | 1.06 |
|  | Cer IIIB 10 μg/mL | 641654 | 450717 | 1.42 |
| IVL | Control | 290481 | 337526 | 0.86 |
|  | PSO-Cer 1 μg/mL | 297560 | 330243 | 0.90 |
|  | PSO-Cer 10 μg/mL | 346991 | 342912 | 1.01 |
|  | Cer IIIB 1 μg/mL | 344267 | 324690 | 1.06 |
|  | Cer IIIB 10 μg/mL | 367128 | 340758 | 1.08 |
| TGM1 | Control | 261091 | 470078 | 0.56 |
|  | PSO-Cer 1 μg/mL | 341619 | 467809 | 0.73 |
|  | PSO-Cer 10 μg/mL | 401546 | 474246 | 0.85 |
|  | Cer IIIB 1 μg/mL | 308361 | 468868 | 0.66 |
|  | Cer IIIB 10 μg/mL | 516985 | 476451 | 1.09 |
| ELOVL4 | Control | 136186 | 511513 | 0.27 |
|  | PSO-Cer 1 μg/mL | 326321 | 506498 | 0.64 |
|  | PSO-Cer 10 μg/mL | 313919 | 516404 | 0.61 |
|  | Cer IIIB 1 μg/mL | 441558 | 511597 | 0.86 |
|  | Cer IIIB 10 μg/mL | 468003 | 513438 | 0.91 |
| CERS3 | Control | 235390 | 413762 | 0.57 |
|  | PSO-Cer 1 μg/mL | 291989 | 403317 | 0.72 |
|  | PSO-Cer 10 μg/mL | 275187 | 394656 | 0.70 |
|  | Cer IIIB 1 μg/mL | 359965 | 392955 | 0.92 |
|  | Cer IIIB 10 μg/mL | 349226 | 391063 | 0.89 |

**Table S3B. Raw densitometric data for Western blot analysis (HDF-α cells, UVB model).**

| Target | Group | Target IDV | ACTIN IDV | Ratio (Target/ACTIN) |
| --- | --- | --- | --- | --- |
| COL1A1 | Control | 309303 | 297236 | 1.04 |
|  | Model (UVB) | 125774 | 289537 | 0.43 |
|  | PSO-Cer 1 μg/mL | 312183 | 304525 | 1.03 |
|  | PSO-Cer 10 μg/mL | 307910 | 293718 | 1.05 |
|  | Cer IIIB 1 μg/mL | 306440 | 307425 | 1.00 |
|  | Cer IIIB 10 μg/mL | 401193 | 307639 | 1.30 |
| ELN | Control | 370442 | 423938 | 0.87 |
|  | Model (UVB) | 194390 | 423808 | 0.46 |
|  | PSO-Cer 1 μg/mL | 326907 | 428386 | 0.76 |
|  | PSO-Cer 10 μg/mL | 369410 | 412073 | 0.90 |
|  | Cer IIIB 1 μg/mL | 368708 | 432088 | 0.85 |
|  | Cer IIIB 10 μg/mL | 366180 | 427431 | 0.86 |

**Table S4. Primer sequences used for qPCR analysis.**

| **Gene** | **Forward Primer (5′–3′)** | **Reverse Primer (5′–3′)** |
| --- | --- | --- |
| CERS3 | ACA TTC CAC AAG GCA ACC ATT G | CTC TTG ATT CCG CCG ACT CC |
| ELOVL4 | TCC TTT GTC TGG CTG CTG GT | TCC TTT GGC CAG GAG AGC TG |
| FLG | TCG GCA AAT CCT GAA GAA TCC AGA | GCT TGA GCC AAC TTG AAT ACC ATC AG |
| CASP14 | GAC CTG GAT GCT CTG GAA CAC A | GAA TCG ATG GCC TGC TGG A |
| BLMH | TGT GGT TTG GCT GTG ATG TT | GCA CCA TCC TGA TCA TCC TT |
| IVL | CCA TCA GGA GCA AAT GAA ACA G | GCT CGA CAG GCA CCT TCT G |
| TGM1 | CCC CGG TTG GCA TAC ACA | GAG CGG AAG GCA GTA GAG ACA |
| COL3A1 | ACT TGG GAT TGC TGG GAT CA | TTT CAC CCT TGA CAC CCT GA |
| COL1A1 | CAC CAA TCA CCT GCG TAC AG | GCA GTT CTT GGT CTC GTC AC |
| ELN | CAG CCA AAT ATG GAG CAG CA | GCT CCC ACT AGG CCA AAC T |
| GAPDH | GTC GGA GTC AAC GGA TTT GG | ATC TCG CTC CTG GAA GAT GG |

**Figures**

**Figure S1**. Representative LC-QTOF MS and MS/MS spectra of major ceramide species identified in PSO-Cer.

(Top panel) Full-scan mass spectrum showing the [M+H]⁺ precursor ions of the major ceramide species detected in enzymatically synthesized PSO-Cer (m/z 556.5270, 578.5119, 580.5290, and 582.5438, corresponding to C16:0, C18:3, C18:2, and C18:1 ceramides, respectively).

(Bottom panel) CID fragmentation spectrum of the C18:3 ceramide precursor ion (m/z 578.5119), with diagnostic fragment ions labeled (m/z 560.50 [M+H-H₂O]⁺, m/z 282.28 [t18:0−2H₂O+H]⁺, and m/z 261.22 [C₁₇H₂₉O]⁺).
